# Pain as a Symptom of Mental Health Conditions Among Undocumented Migrants in France: Results From a Cross-Sectional Study

**DOI:** 10.3389/ijph.2024.1607254

**Published:** 2025-01-06

**Authors:** Sohela Moussaoui, Nicolas Vignier, Stephanie Guillaume, Florence Jusot, Antoine Marsaudon, Jérôme Wittwer, Paul Dourgnon

**Affiliations:** ^1^ PHARes Team, National Institute of Health and Medical Research (INSERM) U1219 Bordeaux Population Health Centre Recherche (BPH), Bordeaux University, Bordeaux, France; ^2^ Institut de Recherche et de Documentation en Économie de la Santé, Paris, France; ^3^ Department of Family Practice, Sorbonne Université, Paris, France; ^4^ Hôpitaux Universitaires Paris Seine-Saint-Denis, Bobigny, France; ^5^ National Institute of Health and Medical Research (INSERM) U1137 Infection, Antimicrobiens, Modélisation, Evolution, Paris, France; ^6^ Mixed Research Unit (UMR) 8007 Laboratoire d’Economie de Dauphine (LEDA), Paris, France

**Keywords:** transients and migrants, pain, anxiety, sleep disorder, depression

## Abstract

**Objectives:**

This study aimed to explore the associations between mental health status and experienced pain among undocumented migrants (UMs) in France.

**Methods:**

We used data from the multicentric cross-sectional “Premier Pas” study conducted in the Parisian and Bordeaux regions from February to April 2019. Participants over 18 years of age were recruited from sixty-three sites. Pain was assessed through two variables: overall pain and musculoskeletal pain. Mental health conditions, including anxiety, sleep disorders, depression, and posttraumatic stress disorder (PTSD) were evaluated. Logistic regression models were used to explore associations, controlling for social determinants of health (SDHs).

**Results:**

Our findings revealed significant associations between mental health status and pain among the 1,188 included participants. Sleep disorder was associated to higher odds of musculoskeletal pain (aOR = 2.53, 95% CI [1.20–5.33], *p* = 0.014). Stratified results indicated that among women, depression was associated to higher odds of pain (aOR = 4.85, 95% CI [1.53–13.36], *p* = 0.007).

**Conclusion:**

This large study confirms the connection between mental health status and pain among UMs, providing valuable evidence for clinicians to address mental health issues in this population.

## Introduction

The term undocumented migrants (UMs) refers to individuals without formal residence recognition, resulting in an irregular migration status [1]. Some UMs may have held temporary permits that have expired, whereas others enter a country without permission or are denied asylum. The exact number of UMs in Europe and France is uncertain, with varying estimates. In France, UMs are prohibited from legal employment, often resorting to informal jobs that lack essential protections and rights [2].

Migrants and individuals in ethnic minority groups experience health disadvantages due to intersecting factors such as racial identity and socioeconomic status, particularly vulnerable groups such as women and sex workers [3].

Research on health disparities between UMs and regular residents (including documented migrants) in Europe is limited. However, evidence shows that UMs face numerous health challenges [4]. Compared with the majority population, UMs report poorer self-perceived health and are at increased risk for mortality from non-communicable diseases, such as cardiovascular issues and cancers [1, 4–9]. High rates of maternal mortality and adverse birth outcomes, including preterm birth and low birth weight, have also been noted among UMs [1, 10]. Furthermore, undocumented women often have limited access to contraception and family planning services, resulting in higher rates of unintended pregnancies and poorer maternal and child health outcomes [1, 11].

Mental health is another critical area, as UMs are more likely to have risk factors for conditions such as depression, anxiety, and posttraumatic stress disorder (PTSD) than both the general population and documented migrants are [1, 4, 12, 13].

In France, UM faces a greater burden of HIV and other infectious diseases, including significant rates of dental infections and chronic hepatitis B [14]. Healthcare for UMs is a polarizing issue in France [15]. The Aide Médicale d’Etat (AME, State Medical Aid) provides means-tested healthcare access, with residence in the country for more than 3 months and low income required for eligibility [16]. Nearly half of eligible individuals do not apply for this program, resulting in limited access [17–20]. AME holders often face discrimination, leading to unmet health needs [21]. Barriers to care increase the likelihood of delayed diagnoses, and while some non-governmental organizations (NGOs) offer support, their capacity for follow-up and preventive care is limited [19, 21–26].

Chronic pain, particularly musculoskeletal pain, contributes significantly to mental illness [27]. A positive association between pain and mental health has been described, although the causal pathway remains unclear [28–31]. Bidirectional relationships exist, with similar risks for developing mental illness due to pain and *vice versa* [32]. Both pain and mental illness share biological mechanisms and brain regions [33, 34]. Behavioral factors can also explain this relationship. For example, the fear–avoidance model suggests that negative thought patterns can lead to fear of movement, perpetuating a cycle of pain, disability, and depression [35]. The relationship between pain and mental health seems to vary according to gender, with the effect being stronger among females [36, 37].

In the general population, social determinants of health (SDHs), such as income or employment status, have been identified as key factors associated with pain [38]. For example, associations between food insecurity and site-specific pain, such as musculoskeletal pain, have been described in the general population [39–41]. Socioeconomically deprived individuals are more likely to experience chronic pain, more severe pain, and a greater degree of pain-related disability [40, 42].

In immigrant populations, associations between mental health and SDHs have been described [2, 43–46]. Poor working conditions are linked to adverse health outcomes and pain in immigrants [2, 43, 47–49].

For UMs in Europe, few studies have provided evidence on the associations between SDHs and mental health [4, 13, 50]**.** Some studies indicate that postmigration housing insecurity is associated with worse mental health in adult UMs [51, 52]. Food insecurity and a longer duration of residence in the host country are also linked to poorer mental health among UMs in the United States (U.S.) [53]. Additionally, the type of work contract may influence health outcomes for UM workers [54]. Conversely, social support and community engagement are protective factors for the mental health of UMs in Europe [51, 52, 55]. Gaining documented status has positive effects on the mental health of UMs in Switzerland [50, 56].

The literature describes associations between mental health and pain in various immigrant groups, with stronger links to conditions such as depression, PTSD or anxiety in immigrant populations than in the general population [46]. Among UMs in the U.S., associations between pain and psychological distress, such as anxiety and depression, have been described [57]. In France, however, data on UMs remain limited, and the relationship between pain and mental health in this group has not been studied.

Immigrant populations face migration-related stressors, such as physical and verbal violence, which worsen the effects of displacement, including limited resource availability and exclusion from citizenship-oriented health policies [58, 59]. The literature describes a high prevalence of depression, anxiety, stress, sleep disorders, and PTSD among immigrants during and after migration [60, 61]. PTSD and depression are particularly prevalent among UMs compared with the general population and are influenced by low socioeconomic status and integration policies [51, 62].

UMs experiencing social isolation, abuse, or financial instability are at increased risk of mental health issues such as anxiety, depression or sleep disorders [50, 52, 58, 63, 64]. Access to psychosocial healthcare is often hindered by a lack of insurance, taboos about mental health, and distrust in practitioners’ abilities to treat mental health conditions [55, 58, 63]. Since UMs may not spontaneously report mental health difficulties, healthcare professionals need to actively inquire about the needs of these individuals [12].

Pain is a frequent reason for consultation, which initiates first contact with the healthcare system in the host country [65, 66]. The literature suggests that pain is often experienced by UMs, with musculoskeletal pain being the most frequently reported type in healthcare settings [58, 67].

UMs often earn an income through manual and physically demanding occupations that can induce, maintain or increase musculoskeletal pain [2, 3, 68].

Among UMs, mental health and pain are often associated with SDHs. It is therefore worth investigating whether the relationship between pain and mental health persists once SDHs are controlled for. We hypothesized that this relationship would persist after controlling for SDHs. Our main objective was to explore whether the association between mental health and pain remained among UMs in France after adjusting for SDHs.

## Methods

### Study Design and Participants

Premier Pas was a multicentric cross-sectional study carried out in the Parisian and Bordeaux regions of France between February and April 2019. This study, which was based on multidisciplinary approaches, sought to better understand the experiences of UMs living in France and to assess their health status and access to rights and healthcare. The inclusion criteria consisted of being undocumented and over 18 years old. The questionnaire was translated into fourteen different languages to interact with participants: French, English, Spanish, Russian, Albanian, Portuguese, Arabic, Dari, Chinese, Tamil, Pashto, Bengali, Pulaar, and Bambara [17–19]. Individuals were recruited from 63 sites that provide support or assistance for individuals experiencing social deprivation and immigrants [19]. The structures included “Espace Solidarité Insertion,” which offers daytime shelter, social and health services for homeless people, various NGOs, and Hospital Health Access Sites (“Permanence d’Accès aux Soins de Santé”). The latter provide social assistance and healthcare to vulnerable populations, but their geographic scope is limited to certain cities. Additionally, another type of structure included was local health insurance centers (“Caisses Primaires d’Assurance Maladie”), which operate at a departmental level and deliver various social subsidies and indemnities for registered beneficiaries for claims ranging from sickness, pregnancy, disability or death. Point of Access to Rights (“Point d’accès aux droits”), which are free and permanent reception sites, provide information and resources for legal or administrative problems and were also included as recruitment sites. Finally, three more recruitment sites were included: public baths, free health centers of Doctors of the World (“CASO”) and maternal and child protection centers (“Protection Maternelle et Infantile”), which is a departmental service that is responsible for protecting the health of mothers and newborns.

### Data Collection

#### Dependent Variables

Data on pain experienced at the time of the study were collected, and the pain experienced included abdominal, musculoskeletal, headache/migraine, and chest or pelvic pain. A binary variable, “all pain,” was created (musculoskeletal or abdominal, or headache/migraine or chest or pelvic pain) from the variables related to the different types of pain. As we were more interested in the experience of pain than its mechanism or cause, this grouping seemed justified and, at the same time, enabled us to increase the power of our analyses.

Owing to the sample size, the comparative analyses were performed only on musculoskeletal pain and on the variable “all pain.”

#### Independent Variables

Mental health was assessed through several questions. The participants were asked if they were currently experiencing various conditions, including mental health conditions. If a participant answered “yes” for a mental health condition, then follow-up questions were asked to clarify whether they were experiencing a sleep disorder or anxiety. For depression, we used the Patient Health Questionnaire-9 (PHQ-9) and a cutoff score of 10 or more to estimate moderate to severe depression [69]. PTSD was assessed with the Primary Care PTSD Screen for DSM-5 (PC-PTSD-5) [70]. A score of three or above was used as the threshold for diagnosing PTSD. Depression and PTSD were assessed with the PHQ-9 and the PC-PTSD-5, respectively, for all participants regardless of their answers to the general question.

Our literature review, described in the introduction, helped identify confounders related to pain and mental health. We categorized these determinants into coherent groups according to the following: demographic characteristics (age and gender), migration characteristics (region of origin, time since arrival in France, motives for migration), social characteristics (current job, job in the country of origin, fluency in French), the family environment (isolation), material living conditions (housing type, food insecurity) and access to care (“Aide Médicale de l’Etat”, AME”). The latter is a specific means-tested publicly funded health insurance for UMs that covers medical expenses at 100% of real costs with some excluded treatments [71].

### Data Analysis

In the first stage, we described the prevalence of different types of pain using percentages and then compared the categories of pain according to different mental health conditions via a chi-square test. The data were weighted on the basis of the probability of inclusion.

In the second step, we performed univariate analyses via logistic regression for musculoskeletal pain and the variable “all pain,” with pain as the dependent variable and mental health as the main independent variable. When an association was significant in the univariate analysis, we performed multivariate analyses. A difference was considered significant when the *p*-value of the crude odds ratio (OR) for the univariate result was <0.200.

To address collinearity and avoid overadjustment in the multivariate analyses, we used a hierarchical regression method. Blockwise selection was used, with pain as the dependent variable and mental health as the independent variable, and the different categories were added step by step. In this six-step process, we added each group of variables sequentially, monitoring how the coefficients changed to ensure that the models remained robust. To avoid weighing down the models and reducing their performance, we added only the variables with a *p*-value of the crude OR of less than 0.200 to the model. Alternatively, within a group of variables for which all the crude ORs were greater than or equal to 0.200, we selected the variable with the smallest *p*-value.

For power purposes, the age categories “18–29 years” and “30–39 years” were grouped together into the category “18–39 years” in some analyses when there were small numbers in one category.

In the final step, analyses were performed to stratify by gender, as the literature has shown that associations between pain and mental health are influenced by gender [36, 37].

The threshold of significance was 0.05 and 95% confidence intervals were used. All analyses were performed with Stata software.

## Results

A total of 1,223 participants were included in the Premier Pas study, and 1,188 answered questions related to current health conditions. Overall, 28.8% of the participants were women, and the mean age was 37.1 years (95% confidence interval (CI) [36.5–37.7]). In terms of geographic origin, 62.5% of the participants were from sub-Saharan Africa, and 23.7% were from North Africa. Approximately half of the individuals in the sample were registered beneficiaries of the AME at the time of the study. A total of 37.8% of the participants had arrived less than 1 year before the time of the survey, and 33.9% had lived in France for at least 3 years. More than half of the participants were fluent in French (56.4%). Regarding material living conditions, 42.5% of the participants had precarious housing, and 30.7% reported often facing food insecurity. Table 1 displays all the characteristics of the population.

**TABLE 1:** Description of the population (Premiers Pas, France, 2019).

	n	% (weighed)
Total	1,223	
Demographic characteristics		
Gender	**1,220**	
Woman	426/1,220	28.8
Man	794/1,220	71.2
Age category	1,216	
18–29	333/1,216	36.2
30–39	443/1,216	33.9
40 or older	440/1,216	29.9
Migration characteristics		
Region of origin	1,209	
North Africa	363/1,209	23.7
Subsaharan Africa	630/1,209	62.5
Other	216/1,209	13.8
Arrival in France	1,215	
Less than 3 months	132/1,215	15.3
3 months −1 year	324/1,215	22.5
1 year −3 years]	314/1,215	28.3
3 years–5 years	147/1,215	11.7
More than 5 years	298/1,215	22.2
Reason for migration	1,036	
Health	121/1,036	8.4
Not health	915/1,036	91.6
Social characteristics		
Current job	1,212	
Yes	286/1,212	21.1
No	926/1,212	78.9
Job in the country of origin	1,214	
Employee and self-employed	540/1,214	44.9
Manual worker	361/1,214	32.3
Student and other	107/1,214	8.1
Does not work/has never worked	206/1,214	14.7
Fluency in French	1,222	
Very good/somewhat good	643/1,222	56.4
Not very good/poor or very bad	277/1,222	21.6
Non-French speaking	302/1,222	22.0
Family environment		
Isolation	1,191	
Alone	475/1,191	41.5
Alone with a child	226/1,191	18.5
In a couple without children	132/1,191	10.7
In a couple with child (ren)	358/1,191	29.3
Material living conditions		
Housing type	1,214	
Ordinary	536/1,214	38.1
Collective	219/1,214	19.4
Precarious	459/1,214	42.5
Food insecurity	1,214	
Often	321/1,214	30.7
Sometimes	448/1,214	36.6
Never	445/1,214	32.7
Access to care		
Aide Médicale d’Etat^a^	1,218	
Yes	526/1,218	43.8
No	692/1,218	56.2

^a^ Healthcare insurance for undocumented immigrants translates as “State Medical Assistance.”

A total of 14.3% of the participants reported experiencing pain (n = 176) regardless of the type, 8.3% (n = 103) reported experiencing musculoskeletal pain, 3.5% (n = 48) reported experiencing abdominal pain, 2.7% (n = 37) reported experiencing headache, 1.2% reported experiencing pelvic pain (n = 25) and 1.8% reported experiencing chest pain (n = 12).

**FIGURE 1:**
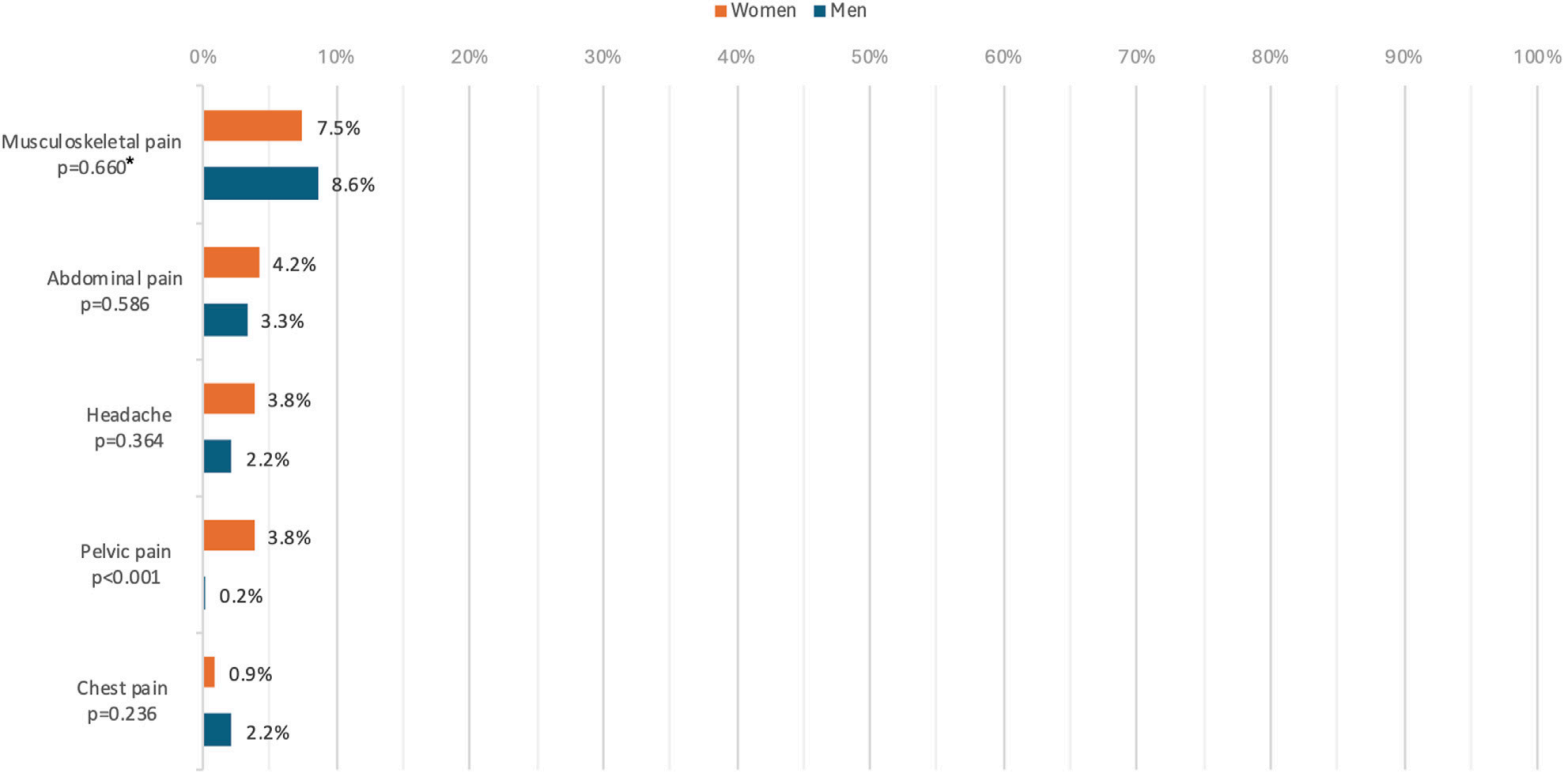
Rates of the different types of pain by gender (Premiers Pas, France, 2019). N = 1118, p*: p-value of the chi-2 test.

No differences were found between women and men except in pelvic pain, for which the rate was greater among women (Figure 1).

In terms of mental health, 11.5% of the participants reported having anxiety (n = 131), 15% reported having sleep disorders (n = 176), 29.5% had moderate to severe depression (n = 384, PHQ-9 score), and 16.2% had PTSD (n = 210, PC-PTSD-5 score).

In terms of gender, no differences were found between women and men. The proportions are reported for the following conditions: anxiety (10.6% n = 50/418 and 11.4%, n = 78/767), sleep disorders (14.5%, n = 72/418 and 15.0%, n = 102/767), depression (32.9%, n = 164/367 and 27.9%, n = 219/663) and PTSD (18.7%, n = 85/409 and 15.0%, n = 123/764).

### Pain and Mental Health

Table 2 presents crude and adjusted associations between pain (all pain and musculoskeletal pain) and mental health. Individuals who were depressed, those with sleep disorder and those with anxiety were more likely to report pain. Individuals with PTSD had significantly higher likelihood of overall pain, at bivariate level and not in multivariate one. No difference was observed between musculoskeletal pain and PTSD.

**TABLE 2:** Prevalence, univariate and multivariate analyses for pain and mental health-related variables, Premiers Pas, France, 2019.

	N	Weighted percentage	All pain					Musculoskeletal pain				
			n (%)	p-value (Chi2)	Univariate analysis (cOR)	Multivariate analysis (aOR, 95% CI) N = 953	*p*-value	n (%)	p-value (Chi2)	Univariate analysis (cOR)	Multivariate analysis (aOR, 95% CI) N = 953	*p*-value
			176/1,188 (14.3)					103/1,188 (8.3)	
Depression	**1,031**		**1,011**	**0.001**		**N = 826**		**1,011**	**0.019**			
Yes	384	29.5	**75/379 (18.0)**		**1.52°**	1.32, [0.66–2.88]	0.389	**45/379 (8.5)**		0.92	—	—
No	647	70.5	**74/632 (12.7)**		ref	**ref**	**ref**	**47/632 (9.1)**		ref	—	—
Sleep disorder	**1,188**		**1,188**	**<0.001**		**N = 959**		**1,188**	**<0.001**		**N = 959**	
Yes	176	15.0	**44/176 (33.0)**		2.67***	2.83*, [1.39–5.76]	**0.040**	**26/176 (16.3)**		2.67*	2.53*, [1.20–5.33]	**0.014**
No	1,012	85.0	**132/1,012 (11.0)**		**ref**	**ref**	**ref**	**77/1,012 (6.8)**		**ref**	**ref**	**ref**
Anxiety	**1,188**		**1,188**	**0.048**		**N = 959**		**1,188**	**<0.001**			
Yes	131	11.5	**27/131 (24.7)**		**2.20°**	1.59, [0.68–3.72]	0.285	**15/131 (8.6)**		1.05	—	—
No	1,057	88.5	**149/1,057 (13.0)**		**ref**	**ref**	**ref**	**88/1,057 (8.2)**		ref	—	—
PTSD	**1,176**		**1,150**	**0.001**				**1,150**	0.183		**N = 937**	
Yes	210	16.2	**46/208 (20.7)**		1.67	—	—	29/208 (12.7)		1.76°	1.31, [0.61–2.79]	0.489
No	966	83.8	**125/942 (13.5)**		ref	—	—	71/942 (7.6)		ref	ref	ref

PTSD, posttraumatic stress disorder.

*p*-value (Chi2): *p*-value using chi-square test. OR, crude odds-ratio; aOR, adjusted odds-ratio; *p*-value: °*p* < 0.20; *p < 0.05; **p < 0.01; ***p < 0.001.

95% CI: 95% Confidence interval. —: variable not included in the final model ref: reference category.

Multivariate analysis: Models after adjusting for mental health and socioeconomic related variables: gender, age, region of origin, time since arrival in France, reason for migration, occupation type, job in the country of origin, fluency in French, isolation, housing type, food insecurity, healthcare insurance with the Aide Médicale de l’Etat. The sample size N = XX is in bold as well as the values when a significant difference was observed.

Multivariate analyses were performed when the univariate association was significant. The multivariate analyses controlling for socioeconomic indicators revealed that sleep disorder was associated with higher odds of overall pain (adjusted odds ratio (aOR) = 2.83, 95% CI [1.39–5.76], *p* = 0.040). Sleep disorder was also associated with higher odds of musculoskeletal pain (aOR = 2.53, 95% CI [1.20–5.33], *p* = 0.014).

### Stratified Analyses by Gender

As shown in Table 3, among women, those with depression had significantly higher odds of overall pain after controlling for socioeconomic variables (aOR = 4.85, 95% CI [1.53–13.36], *p* = 0.007). No associations were found between mental health and musculoskeletal pain among women.

**TABLE 3:** Univariate and multivariate analyses for musculoskeletal pain, abdominal pain and headache with mental health-related variables, stratified by gender (Premiers Pas, France, 2019).

	Women							
	All pain				Musculoskeletal pain			
	Univariate analysis	Multivariate analysis n = 548			Univariate analysis	Multivariate analysis n = 548		
	OR	ORa	95% CI	*p*-value	OR	aOR	95% CI	*p*-value
Depression	**N = 362**	**N = 296**			**N = 362**	**N = 257**		
Yes	3.38**	4.85**	**[1.53–13.36]**	**0.007**	4.90**	3.59°	[0.93–13.82]	0.063
No	ref	ref	ref	ref	ref	ref	ref	ref
Sleep disorder	**N = 418**	**N = 343**			**N = 418**	**N = 297**		
Yes	4.09**	2.54°	[0.91–7.11]	0.076	3.16*	1.70	[0.35–8.31]	0.509
No	ref	ref	ref	ref	ref	ref	ref	ref
Anxiety	**N = 418**	**N = 343**			**N = 418**	**N = 297**		
Yes	5.72**	2.33°	[0.92–5.88]	0.074	5.10**	2.94°	[0.87–9.92]	0.083
No	ref	ref	ref	ref	ref	ref	ref	ref
PTSD	**N = 404**	**N = 332**			**N = 404**	**N = 286**		
Yes	1.83°	1.50	[0.54–4.16]	0.440	2.42°	1.52	[0.46–5.05]	0.496
No	ref	ref	ref	ref	ref	ref	ref	ref

	Men							
	All pain				Musculoskeletal pain			
	Univariate analysis	Multivariate analysis n = 548			Univariate analysis	Multivariate analysis n = 628		
	OR	aOR	95% CI	*p*-value		aOR	95% CI	*p*-value
Depression	**N = 648**				**N = 648**	**N = 533**		
Yes	1.06	—	—	—	0.42°	0.28°	[0.05–1.47]	0.131
No	ref	—	—	—	ref	ref	ref	ref
Sleep disorder	**N = 767**	**N = 616**			**N = 767**	**N = 616**		
Yes	4.05**	2.80*	**[1.05–7.48]**	**0.040**	2.57°	7.14*	**[1.60–31.87]**	**0.010**
No	ref	ref	ref	ref	ref	ref	ref	ref
Anxiety	**N = 767**				**N = 767**	**N = 616**		
Yes	1.53	**—**	**—**	**—**	0.35°	0.12*	**[0.02–0.82]**	**0.031**
No	ref	—	—	—	ref	ref	ref	ref
PTSD	**N = 743**				**N = 743**			
Yes	1.64	—	—	—	1.57	—	—	—
No	ref	ref	ref	ref	ref	ref	ref	ref

PTSD, Posttraumatic stress disorder *p*-value (Chi2): *p*-value using chi-square test.

OR, crude odds-ratio; aOR, adjusted odds-ratio; *p*-value: °*p* < 0.20; *p < 0.05; **p < 0.01; ***p < 0.001.

95% CI, 95% Confidence interval. —: variable not included in the final model ref: reference category.

Multivariate analysis: Models after adjusting for mental health and socioeconomic related variables: gender, age, region of origin, time since arrival in France, reason for migration, occupation type, job in the country of origin, fluency in French, isolation, housing type, food insecurity, healthcare insurance with the Aide Médicale de l’Etat. The sample size N = XX is in bold as well as the values when a significant difference was observed.

Among men, those with sleep disorder had significantly higher odds of overall pain after controlling for socioeconomic variables (aOR = 2.80 95% CI [1.05–7.48], *p* = 0.040). Sleep disorder (aOR = 7.14, 95% CI [1.60–31.87], *p* = 0.010) and anxiety (aOR = 0.12, 95% CI [0.02–0.82], *p* = 0.031) were associated with musculoskeletal pain.

## Discussion

### Main Results

Overall, clear associations between several mental health conditions and pain were revealed after controlling for SDHs. These results are consistent with the international literature [62, 72]. Similar to our study, a Swedish study revealed that a higher risk of chronic and severe pain among immigrants was significantly influenced by depression [73]. In another cohort study of Syrian refugees in Norway 12 months after their arrival in Northern Europe, poor mental health was a predictor of chronic pain at follow-up [72]. Finally, studies also support that trauma and pain, especially joint pain, are connected [74].

The rationale for including the “all pain” variable was that our study addressed the experience of pain and its relationship with mental health. The experience of pain, rather than its mechanism or cause, was the focus of this work, and this grouping made sense to us. Moreover, the inclusion of this variable increased the power of our analyses. This grouping and the results are also coherent from the perspective of screening UMs who visit clinics for pain. When UMs visit a clinic because they are experiencing pain, they should be screened for mental health problems, as they are more at risk.

### Gender Stratification

We stratified the data by gender to verify whether the relationship between mental health and pain varied according to gender on the basis of the literature [36, 37]. Our results confirmed this hypothesis, as some associations remained only for women or men. Depression was associated with higher odds of overall pain among women, and sleep disorder was associated with higher odds of overall pain and musculoskeletal pain among men. The associations found in our study were in line with those described in the literature in the general population [36, 37, 75].

Surprisingly, after controlling for socioeconomic indicators, a negative association appeared for men. Anxiety was associated with lower odds of musculoskeletal pain among men. The association usually reported in the literature is that anxiety is a risk factor for musculoskeletal pain [76]. Nevertheless, among veterans, it appears that when anxiety is associated with PTSD, pain perceptions can be reduced [74]. In our study, no association between anxiety and PTSD was found, possibly because of a lack of power. Some associations, such as that between sleep disorder and musculoskeletal pain, among women have been described in the literature but were not found in our population [36, 77].

In addition to musculoskeletal pain and headaches, men had lower rates of reported pain than women did. One reason for this result could be the variation in pain verbalization depending on gender. Pain may be less easily expressed by men not because of biological differences but because of gender performativity [78]. Moreover, the verbalization of anxiety also appears to be gender biased. Male participants appear to be less inclined to express their anxiety but would be more comfortable doing so in certain contexts, such as in online forums or to women in their close circle (to their spouse, mother, etc.) [79].

### Social Determinants of Health

In our study, the multivariate models were adjusted for SDHs. The role of SDHs in pain has been described, and migration seems to play a role [39, 58, 80, 81]. However, how migration affects the association between pain and mental health is unclear. The allostatic load theory explains how migration-related exposures play a role in the accumulation of somatic damage and increase the risk of several diseases [82, 83]. Migration-related stressors such as poor safety, a poor physical environment or poor access to healthcare are associated with chronic pain among immigrants [72].

### Mutual Maintenance

Our results highlight the importance of identifying when pain is experienced by UMs not only because of its prevalence but also because it can be associated with mental health conditions. Our study was observational, so a causal pathway cannot be established between pain and mental health. However, it seems that they can both maintain each other mutually. The “mutual maintenance theory” suggests that chronic pain may exacerbate and maintain PTSD, whereas PTSD may exacerbate and maintain chronic pain [84]. This mutual maintenance occurs through specific mechanisms, such as reminders of trauma, depression and reduced activity levels. This relationship seems to extend further and has an impact on treatment for both mental health conditions and pain. Physical pain may interfere with a patient’s ability to respond to treatment, and the presence of a mental health condition could also interfere with effective pain management [74].

### Perspectives

The pain expressed by the participants was self-assessed, and the causes of pain were not explored. Several underlying hypotheses may explain the cause of pain. The pain experienced could be linked to an unperceived or undiagnosed health problem, and reduced access to healthcare may increase the risk of delayed diagnosis or even non-diagnosis of the cause of pain [26]. In cases where pain can be explained by a physical or organic cause, whether a mental health problem exists must still be investigated, as it may accompany or even maintain the pain [84]. There are situations where pain cannot be medically explained and may either be part of a psychiatric syndrome or remain medically unexplained [85]. Regardless of the cause of the pain, its treatment can be challenging. Few studies have evaluated the impact of pain treatment on reported pain and mental health among immigrants [86, 87]. A study conducted in Norway among refugees from Syria aimed to assess two different interventions to reduce pain and posttraumatic symptoms: the “Physiotherapy Activity a pain Awareness Intervention” (PAAI), which consists of a combination of psychomotor and general physiotherapy, and the “Teaching Recovery Technique” (TRT), which involves cognitive behavioral therapy [86]. The PAAI had no effect on either chronic pain or mental health symptoms after 8 and 12 weeks. One reason that the authors proposed was that the intervention was not sufficiently adapted for the participants and their situations. A large proportion of participants in the intervention group could not attend the sessions. The qualitative analyses revealed that it was difficult to combine the sessions with other activities of daily life, thereby potentially contributing to additional stress [88]. The TRT significantly improved general mental health but did not help reduce chronic pain [89].

### Strengths

The key strength of this study lies in its recruitment of a diverse population and its focus on mental health issues within this vulnerable group. Moreover, to our knowledge, our study is the first to focus on UMs not exclusively recruited in healthcare settings in France. Very little is known in terms of the public health of this population. Thus, this study is highly important, as it provides insights into this hard-to-reach and hard-to-study population. One of the factors that enabled recruitment was the use of diverse languages and the inclusion of numerous sites outside care settings. Even though the sample size was not large, the results of this study are still substantial if we consider that this population is difficult to approach, especially outside healthcare settings. The results presented in this article are the first to document the relationship between pain and mental health in this population.

### Limitations

Unfortunately, the lack of power did not allow us to further explore the implications of PTSD and depression on the different types of reported pain. Additionally, the small sample size of women did not allow us to explore associations between mental health and musculoskeletal pain. For some results, such as the association between sleep disorder and musculoskeletal pain, the CIs were large. Thus, even if the value was significant, the uncertainty was high, and the estimate was less precise because of the size of the 95% CI. A larger sample size might have reduced this uncertainty.

Furthermore, our study does not represent all UMs across France because recruitment took place in two regions. However, our results are representative, as UMs are geographically concentrated in urban regions, particularly in the Parisian region [90]. Future research with larger samples should thoroughly investigate immigration-related factors, including a wide range of sociodemographic and health-related factors that may contribute to health status among immigrants.

Pain could have been studied in more detail, for example, by examining the intensity of the pain or by asking how the pain affected the participants’ lives. Self-assessment of subjective health, such as pain, is influenced by culture, as expectations and norms for a given health dimension can be very heterogeneous [91]. As a result, the subjective self-assessment of pain in this study did not allow us to compare subjective health states between individuals, especially to compare samples from different countries or regions. This can lead to serious misunderstandings about the health status of populations, particularly those that are the most economically disadvantaged. This bias, known as the “DIF” (Differential Item Functioning) effect, can be corrected via more advanced methods, such as “Anchoring Vignettes,” which can be used to detect and correct possible comparability problems. This method was not used in our study, as the study design was not initially intended to study pain in depth. Future studies could be carried out using this type of method to better study pain.

The PHQ-9 and PC-PTD-5 were used in different languages. In this study, interviews were conducted in French (75%), Arabic (8%), English (7%), Spanish (4%), Russian (2%), and Portuguese (2%), accounting for 99% of the questionnaires collected [19]. Although the PHQ-9 has been validated in these languages, this was not the case for the PC-PTSD-5 [92–96]. The PHQ-9 has been validated in French, Arabic, English, Spanish, Russian and Portuguese. The PC-PTSD-5 has only been validated in English but not in French or other languages [70]. For those languages, no suitable and validated scale to measure PTSD symptoms was found in the literature. Thus, PTSD symptoms may have been over- or underestimated in our study, leading to measurement bias for this indicator.

Since our study did not aim to establish causality, the lack of specific time frames does not undermine the relevance of our findings. Moreover, recall bias would have been a challenge. The PHQ-9 and PC-PTSD-5 were administered to all individuals, and anxiety and sleep disorders can rarely be ignored, as these symptoms often have a major impact on quality of life. Thus, the risk of over- or underestimating the prevalence of mental health conditions in our study was reduced. Moreover, since our study was essentially conducted outside care settings, the risk of overestimating mental health conditions if the participants were seeking health services also remained low.

### Conclusion

Our study confirms the link between mental health and perceived pain in this large sample of UMs in France. These results could have a direct impact on the care provided to UMs. This study encourages practitioners to systematically ask questions pertaining to mental health when a patient is experiencing pain to implement targeted interventions, especially when the pain remains medically unexplained. The results call for more specific studies on the associations between certain types of pain and certain mental disorders and the development of treatment algorithms that consider the social and psychological context of UMs.

## Data Availability

The raw data supporting the conclusions of this article will be made available by the authors upon request.
